# A case of intra-articular fasciitis in the elbow joint

**DOI:** 10.1016/j.ijscr.2019.05.024

**Published:** 2019-05-28

**Authors:** Osamu Nakamura, Yoshio Kaji, Yoshiki Yamagami, Tetsuji Yamamoto

**Affiliations:** Department of Orthopaedic Surgery, Kagawa University Faculty of Medicine, 1750-1 Ikenobe, Miki-cho, Kita-gun, Kagawa 761-0793, Japan

**Keywords:** Intra-articular nodular fasciitis, Elbow joint, Magnetic resonance imaging, Histological examination

## Abstract

**Introduction:**

Nodular fasciitis is a benign myofibroblastic proliferation arising from the fascia. Until now, there have been only two reported cases of intra-articular nodular fasciitis in the elbow joint.

**Presentation of case:**

We report a case of a 19-year-old woman with a 3-month history of pain in the left elbow. Contrast-enhanced T1-weighted magnetic resonance imaging (MRI) showed an intra-articular lobulated mass on the anterior portion of the elbow joint, with accompanying effusion. The patient subsequently underwent arthroscopic excision of the mass. Histologically, intra-articular nodular fasciitis was the final diagnosis. At the most recent follow-up, 20 months after surgery, the patient had no subjective symptoms, including pain. The final MRI findings showed no tumor recurrence.

**Discussion:**

As nodular fasciitis is not generally known to arise within a joint, the occurrence at such anatomical locations may lead to a misdiagnosis. Intra-articular nodular fasciitis is rarely encountered, and therefore, is not usually considered during the clinical investigation of joint symptoms.

**Conclusion:**

Preoperative diagnosis was difficult in this case because of nonspecific preoperative clinical findings. Although histological examination is necessary to establish a diagnosis, we recommend that intra-articular nodular fasciitis should be included in the differential diagnosis of intra-articular mass lesions.

## Highlights

• Intra-nodular fasciitis is very rare and there are only two reported cases of intra-articular nodular fasciitis in the elbow joint.
• It is difficult to diagnose preoperatively, so a clear diagnosis can be obtained only after excisional biopsy.
• As local recurrence has not occurred in previously reported cases, we think that arthroscopic resection is suitable for treatment of this disease.

## 1 Introduction

Nodular fasciitis is a benign, usually self-limiting, myofibroblastic proliferation arising from the fascia with a predilection for the upper extremities, trunk, and the head and neck region in young adults [1]. However, it is rare for the lesion to arise in a location inside the joint [2]. Until now, there have been only two reported cases of intra-articular nodular fasciitis in the elbow joint. This report describes a rare case of intra-articular nodular fasciitis arising in the elbow joint. This manuscript is written in accordance with the Surgical CAse REport (SCARE) guidelines [3].

## 2 Presentation of case

The patient was a 19-year-old woman with a 3-month history of pain in the left elbow. She had slept on her left elbow, using it as a pillow, in March 2017. Upon waking up, she could not stretch her left elbow. Thereafter, she forcibly extended her elbow, which resulted in severe pain. She visited a clinic for consultation and was referred to our hospital in June 2017.

On physical examination of the left elbow, mild swelling was detected but no mass was palpated. The range of motion (ROM) of the elbow joint was limited to 120-40°.

**Fig. 1:**
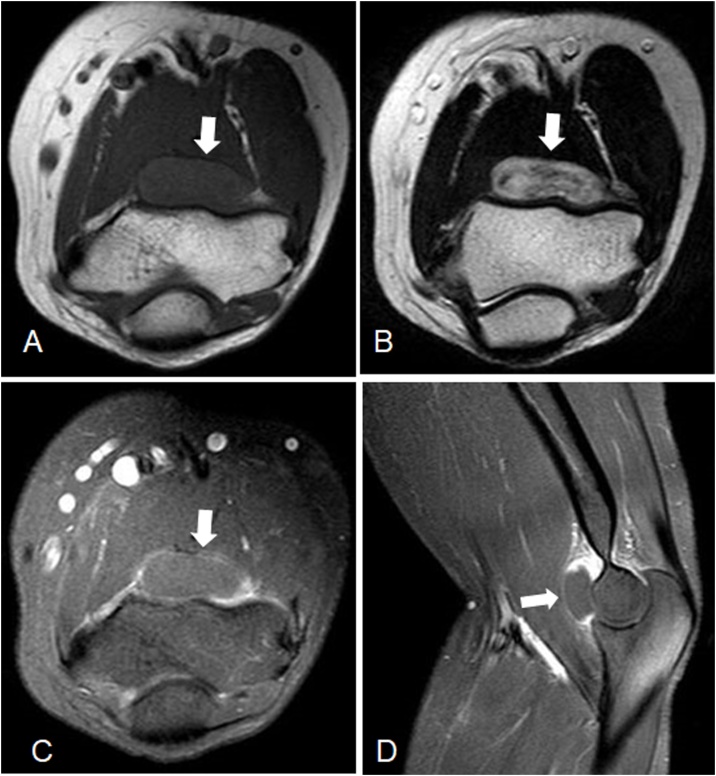
Preoperative axial T1-weighted (A) and axial T2-weighted (B) magnetic resonance (MR) images. Contrast-enhanced T1-weighted axial (C) and sagittal (D) MR images.

Plain radiographs of the left elbow showed no calcification or medullary lesions. Magnetic resonance imaging (MRI) was performed using a 1.5-T MR scanner (Philips Healthcare, Best, The Netherlands). There was an intra-articular oval mass measuring 10 mm × 20 mm on the anterior aspect of the distal humerus. The mass seemed to have iso- to slightly high signal intensity compared to the surrounding normal muscle on T1-weighted MRI and a high signal intensity on T2-weighted MRI (Fig. 1A, B). Contrast-enhanced MRI was performed after intravenous injection of gadolinium diethylenetriamine pentaacetic acid. The contrast-enhanced T1-weighted MRI scans showed an intra-articular lobulated mass on the anterior portion of the elbow joint with accompanying effusion (Fig. 1C, D). There were no abnormal findings on peripheral blood examination. These preoperative clinical and imaging findings suggested an initial diagnosis of soft tissue tumor, such as intra-articular pigment villonodular synovitis (PVNS) or intra-articular hemangioma.

**Fig. 2:**
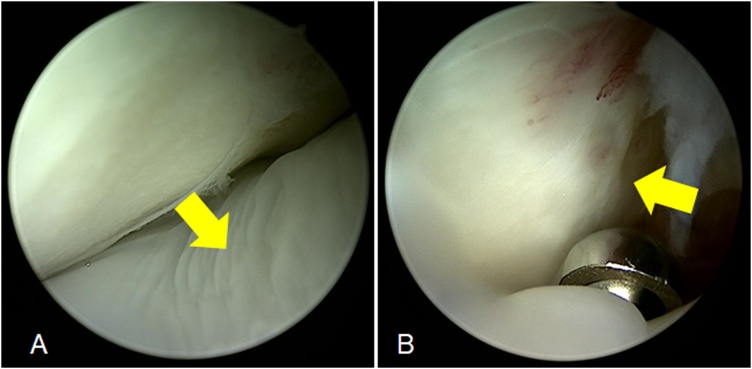
Arthroscopic findings in the patient’s elbow joint. There was a soft, white mass (A) (yellow arrow). The mass was excised piece by piece using an arthroscopic shaver system (B).

The patient subsequently underwent excision of the mass arthroscopically. Before excision, arthroscopic evaluation was performed. The arthroscopic findings revealed that there was a soft, white mass with a smooth surface, which was adherent to the anterior capsule of the elbow joint (Fig. 2A). The mass was excised in fragments using an arthroscopic shaver system (Fig. 2B).

**Fig. 3:**
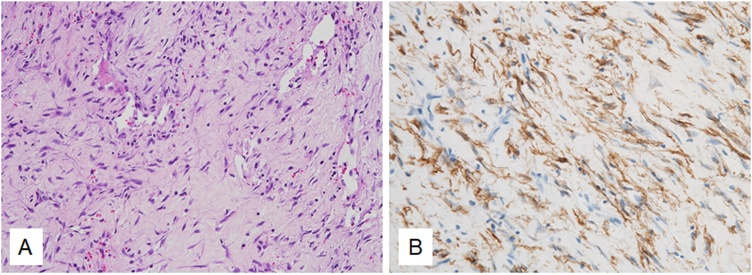
H&E- (A) (magnification, ×40) and anti-α-SMA- (B) (magnification, ×40) stained sections of the excised tumor tissue for histological examination.

**Fig. 4:**
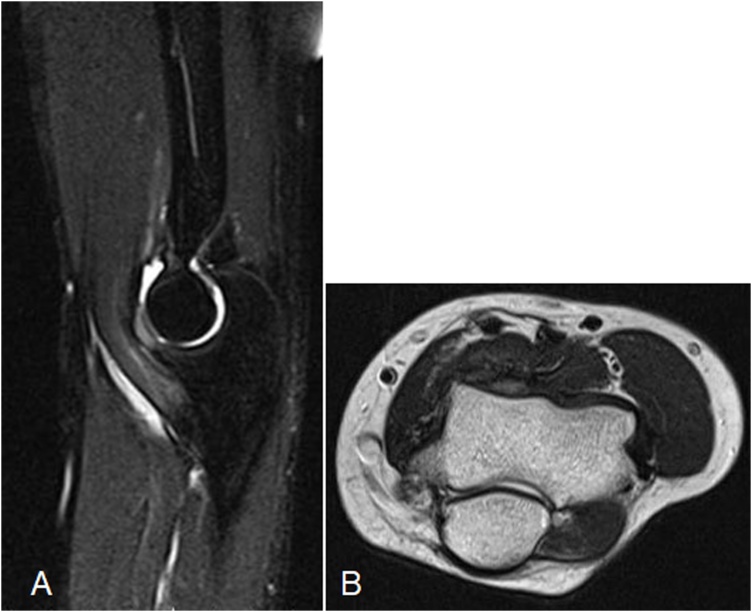
Final axial T1-weighted (A) and sagittal (B) magnetic resonance images.

Histological examination of the excised tumor tissue was performed with hematoxylin-eosin (H&E) staining and it was stained with anti-α-smooth muscle actin (SMA). Histologically, the lesions consisted of myofibroblasts with a myxoid matrix. A few lymphocytes were visible. However, there was no significant nuclear atypia (Fig. 3A). Immunohistochemically, the spindle cells were diffusely positive for α-SMA (Fig. 3B). The overall features were those of intra-articular nodular fasciitis. At the most recent follow-up, 20 months after surgery, the patient had no subjective symptoms, including pain. The ROM of the elbow joint was extended to 150-0°, with no limitations. The final MRI findings showed no tumor recurrence (Fig. 4A, B).

## 3 Discussion

Although the cause is uncertain, nodular fasciitis is likely to be induced by local injury or local inflammatory process. In our patient, there was minor trauma resulting from stretching of the elbow joint. For many patients with nodular fasciitis, the mass grows rapidly and extends for a few weeks [4]. As nodular fasciitis is not generally known to arise within a joint, the occurrence at such anatomical locations may lead to a misdiagnosis [5]. To the best of the authors’ knowledge, the first report of intra-articular nodular fasciitis, published by Van Royen et al. in 1993 [6], presented a case that occurred in the temporomandibular joint. Patients with intra-articular nodular fasciitis typically complain of pain, swelling, restriction of joint motion, and a palpable mass around the joint [7]. In this case, the patient had the same symptoms on her left elbow joint. On literature search, only two other cases of intra-articular fasciitis in the elbow joint were identified [1,8].

A definitive diagnosis of this tumor could not be determined prospectively in this case. The differential diagnosis included intra-articular PVNS, juxta-articular myxomas, synovial chondromatosis, lipoma arborescence, fibroma of the tendon sheath, ganglion cyst, desmoid tumor, and hemangioma. Intra-articular nodular fasciitis is rarely encountered, and therefore, is not usually considered during the clinical investigation of joint symptoms [9].

The features of nodular fasciitis shown by computed tomography and MRI have been reported to be nonspecific [2,[6], [7], [8]]. Nodular fasciitis shows iso- to slightly high-signal intensity on T1-weighted images and high-signal intensity on T2-weighted images by MRI findings. It is difficult for clinicians to differentiate nodular fasciitis from other tumors or tumor-like lesions, including malignancy, solely by imaging analysis, so biopsy or surgical excision is indicated for intra-articular nodular fasciitis for accurate diagnosis [7]. Therefore, biopsy examination is essential to establish the diagnosis [4]. In this case, a clear diagnosis could be made only after excisional biopsy.

Histologically, nodular fasciitis consists of fibroblastic and/or myofibroblastic cell proliferation in the abundant collagenous stroma. Occasionally focal areas of myxoid or hyalinized stroma are observed. Immunohistochemically, the cells stain positively for α-SMA, but negatively for desmin, suggesting focal smooth-muscle cell differentiation [4].

The clinical findings (site, follow up, and recurrence) of intra-articular fasciitis are summarized in Table 1 from 17 case reports [1,2,4,6,7,[10], [11], [12], [13], [14], [15], [16], [17], [18], [19]]. The prognosis of the condition is excellent and local recurrence of the lesion was not observed among the previously reported cases. In contrast, Yamamoto et al. have presented a very rare case of secondary aneurysmal bone cyst in the distal humerus after resection of intra-articular nodular fasciitis within the elbow joint. Intra-articular nodular fasciitis and aneurysmal bone cyst seem to belong to the same biological spectrum defined as USP6-induced tumors according to the report [8]. However, such a case was very rare, and the secondary aneurysmal bone after resection of intra-articular nodular fasciitis was only observed in one of the 17 case reports.

**Table 1:** Clinical features of cases of intra-articular fasciitis.

Authors	Year	Age/ Sex	Site	Treatment	Follow up	Recurrance
Van Royen C, et al.	1993	36/ F	Temporomandibular joint	Open excision	1yr. 6mo	No
Yamamoto T, et al.	2001	49/ M	Knee	Open excision	2yr. 6mo	No
Soejima T, et al.	2003	52/ M	Knee	Arthroscopic excision	2yr	No
Lädermann A, et al.	2008	15/ M	Shoulder	Arthroscopic excision	6mo	No
Nishioka N, et al.	2009	25/M	Elbow	Open excision	1yr	No
Hagino T, et al.	2009	24/ M	Knee	Arthroscopic excision	1yr	No
Harish S, et al.	2011	26/ M	Shoulder	Open excision	6mo	No
Matsuzaki, et al.	2012	20/ M	Knee	Open excision	1yr	No
Ko PY, et al.	2013	4/ F	Knee	Open excision	1yr	No
Gans I, et al.	2014	13/ M	Knee	Arthroscopic excision	–	No
Chan MF, et al.	2014	17/ M	Knee	Arthroscopic excision	1yr. 5mo	No
Yamamoto M, et al.	2015	13/M	Elbow	Open excision	1yr	Secondary aneurysmal bone cyst
Tajima S, et al.	2015	54/ F	Shoulder	Arthroscopic excision	3mo	No
Miyama A, et al.	2018	30/F	Knee	Arthroscopic excision	1yr. 3mo	No
	2018	56/ F	Knee	Arthroscopic excision	1yr. 1mo	No
Choughri, et al.	2018	54/ M	Finger	Open excision	1yr	No
Wang W, et al.	2019	20/ M	Knee	Arthroscopic excision	6mo	No
This case	2019	19/ F	Elbow	Arthroscopic excision	1yr. 8mo	No

In this case, the mass was excised in fragments using an arthroscopic shaver system, so it was not completely resected. Fortunately, there was no recurrence at the final follow-up 20 months after surgery. Nevertheless, the limitation of this case is that the long-term results have not been evaluated. Therefore, it is necessary to follow up this case in the future.

## 4 Conclusion

We reported a rare case of intra-articular fasciitis in the elbow joint. It was difficult to diagnose preoperatively because preoperative clinical findings were nonspecific. Although histological examination is necessary to establish the diagnosis, we recommend that intra-articular nodular fasciitis should be included in the differential diagnosis of intra-articular mass lesions.

## Conflicts of interest

The authors have no conflict of interest.

## Sources of funding

This research did not receive any specific grant from funding agencies in the public, commercial, or not-for-profit sectors.

## Ethical approval

In our case report was not made no experimentation, you just described our clinical practice.

## Consent

Written informed consent was obtained from the patient for publication of this case report and accompanying images. A copy of the written consent is available for review by the Editor-in Chief of this journal on request.

## Author contribution

Osamu Nakamura: performed the surgery; designed this study; writing of the paper.

Yoshio Kaji: assistant to writing of the manuscript.

Yoshiki Yamagami: literature review.

Tetsuji Yamamoto: participated in the critical revision of the article.

## Registration of research studies

My UIN is research registry 4671.

## Guarantor

All authors have read and approved the manuscript and accept full responsibility for the work.

## Provenance and peer review

Not commissioned, externally peer-reviewed.
